# Estimating the number of UK stroke patients eligible for endovascular thrombectomy

**DOI:** 10.1177/2396987317733343

**Published:** 2017-10-04

**Authors:** Peter McMeekin, Philip White, Martin A James, Christopher I Price, Darren Flynn, Gary A Ford

**Affiliations:** 1 Institute of Health and Society, Newcastle University, Newcastle Upon Tyne, UK; 2 Faculty of Health and Life Sciences, 5995 Northumbria University, UK; 3 Institute of Neuroscience (Stroke Research Group), Newcastle University, Newcastle Upon Tyne, UK; 4 NIHR Collaboration for Leadership in Applied Health Research and Care for the South West Peninsula (PenCLAHRC), Exeter, UK; 5 Oxford University Hospitals NHS Trust and Oxford University, UK

**Keywords:** Thrombectomy, ischemic stroke, advanced imaging, service planning

## Abstract

**Introduction:**

Endovascular thrombectomy is a highly effective treatment for acute ischemic stroke due to large arterial occlusion. Routine provision will require major changes in service configuration and workforce. An important first step is to quantify the population of stroke patients that could benefit. We estimated the annual UK population suitable for endovascular thrombectomy using standard or advanced imaging for patient selection.

**Patients and methods:**

Evidence from randomised control trials and national registries was combined to estimate UK stroke incidence and define a decision-tree describing the endovascular thrombectomy eligible population.

**Results:**

Between 9620 and 10,920 UK stroke patients (approximately 10% of stroke admissions) would be eligible for endovascular thrombectomy annually. The majority (9140–9620) would present within 4 h of onset and be suitable for intravenous thrombolysis. Advanced imaging would exclude 500 patients presenting within 4 h, but identify an additional 1310 patients as eligible who present later.

**Discussion:**

Information from randomised control trials and large registry data provided the evidence criterion for 9 of the 12 decision points. The best available evidence was used for two decision points with sensitivity analyses to determine how key branches of the tree affected estimates. Using the mid-point estimate for eligibility (9.6% of admissions) and assuming national endovascular thrombectomy coverage, 4280 patients would have reduced disability.

**Conclusion:**

A model combining published trials and register data suggests approximately 10% of all stroke admissions in the UK are eligible for endovascular thrombectomy. The use of advanced imaging based on current published evidence did not have a major impact on overall numbers but could alter eligibility status for 16% of cases.

## Introduction

Endovascular thrombectomy (EVT) is an effective treatment for acute ischemic stroke with or without intravenous alteplase.^1–8^ The HERMES^9^ individual patient meta-analysis found that for every five patients treated with EVT, two would have reduced disability by at least one level on the modified Rankin Scale (mRS). However, providing EVT presents major challenges in many health care systems. The procedure is typically carried out by neuro-interventionists with anaesthetic support, and requires an infrastructure capable of rapidly performing computed tomography angiography (CTA), with or without advanced imaging (AI) by perfusion-computed tomography (CTP), magnetic resonance imaging (MRI) techniques or CTA collateral scoring (CTA-CS). In clinical trials, CTA alone was generally used to select patients within 6 h of onset, whereas AI techniques were used beyond and sometimes before a 6-h window. The additional infrastructure demands for EVT create the need for a more centralised model of hyperacute stroke care, and robust activity estimates are required for accurate planning to inform service reconfiguration.

In seeking to estimate the anticipated annual demand for this treatment in the UK, we developed a decision tree to estimate the proportion of all stroke patients eligible for EVT, regardless of geographic or service constraints such as non-existent care pathways or a lack of imaging and EVT facilities.

## Patients and methods

Using national registry data from the prospective Sentinel Stroke National Audit Programme (SSNAP) for England, Wales and Northern Ireland,^10^ and adjusted for Scotland using data from the Scottish Stroke Care Audit (SSCA),^11^ we estimated the number of patients hospitalised annually with acute stroke. A decision tree was constructed based upon key inclusion and exclusion criteria from published trials: stroke type, severity, presence of anterior or posterior large artery occlusion (LAO), onset time, pre-stroke disability, the extent of ischemia on CT (or MRI), pre-EVT recanalisation and optional AI. These criteria were applied consistently irrespective of eligibility for intravenous thrombolysis (IVT). The distributions for stroke severity and onset time were extracted from two large UK stroke services. The final decision tree has 12 steps and includes pathways using AI within and beyond 6 h after stroke onset. We did not include basilar artery occlusions presenting after 12 h, as quantifying these at a national level is imprecise. We undertook sensitivity analyses of key decision points to determine the effect upon estimates (proportion of LAO cases, clinical severity, onset time to presentation and core volume).

## Results

### Estimating annual stroke admissions in the UK

**Figure 1.**
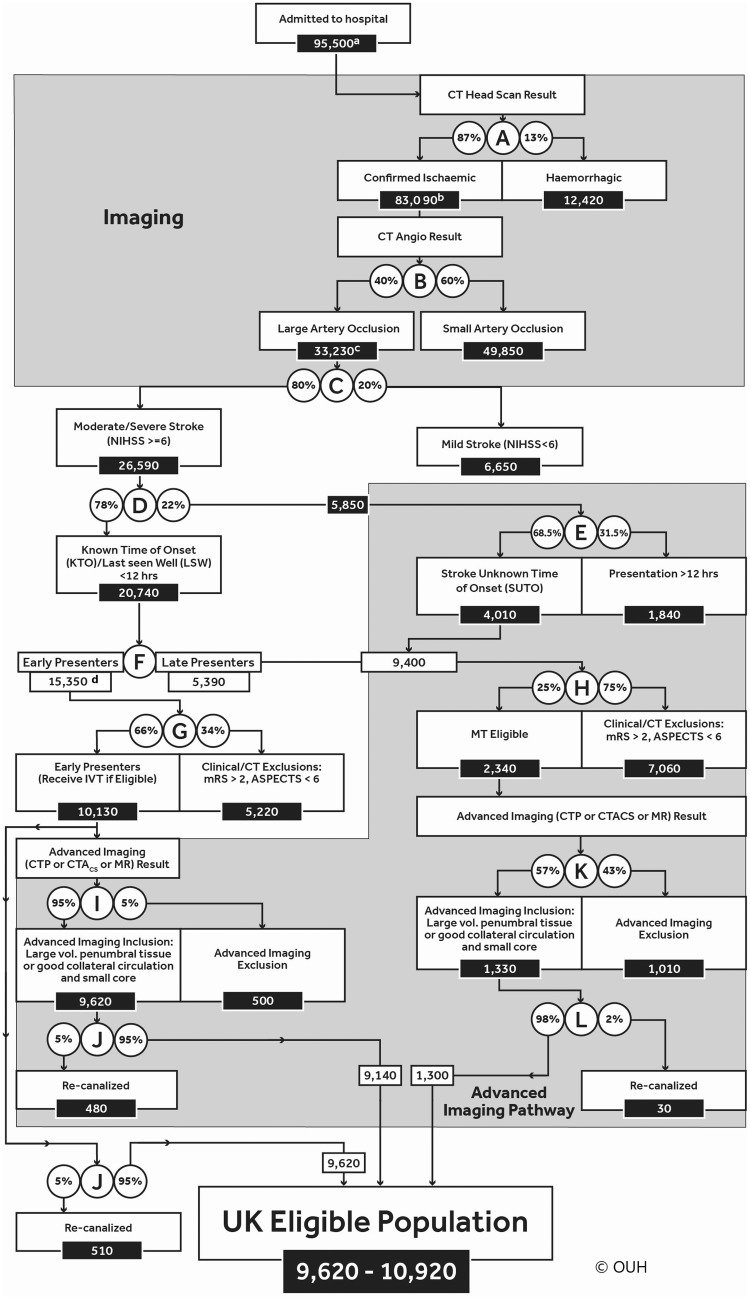
Eligible population ((a) Total UK population including those deemed to be geographically inaccessible. (b) Confirmed infarcts, excluding ∼2% of patients whose status is unconfirmed. (c) Includes basilar artery occlusions eligible for treatment if presenting within 12 h. Others are assumed eligible unless they meet any subsequent exclusion. (d) ‘Early presenters’ – those presenting within 4 h.) *Note*: Patients within the large lower grey shaded box are all dealt with by AI (9400 + 10,130) those who are early presenters (10,130 on the left-hand side) can bypass that step.

The decision tree is presented in Figure 1. It begins with an estimate of annual UK stroke admissions derived from SSNAP and SSCA. SNNAP coverage is comprehensive, with over 80,000 admissions recorded in 2015 from 100% of acutely-admitting hospitals. Case ascertainment in SNNAP is over 98% in England when verified against Hospital Episode Statistics, with the majority of cases omitted being sub-acute or otherwise ineligible for acute intervention. Scaling up this figure by the populations of Wales and Northern Ireland added 4480 and 2240 admissions, respectively. With 8700 admissions from the SSCA, total stroke admissions (excluding subarachnoid haemorrhage) for the UK are 95,500.

### Eligibility by stroke type, location and severity

SSNAP^10^ and SSCA^11^ data report that 13% and 12% stroke admissions respectively are due to intracerebral haematoma. The proportion of ischemic strokes caused by LAO was observed at approximately 41% by the Screening Technology and Outcome Project in Stroke Study (STOP-Stroke), a prospective imaging-based study of stroke outcomes,^12^ and in the trials contributing to the HERMES meta-analysis.^9^ This is supported by a recent UK study of 263 patients reporting a 39% LAO rate.^13^

‘Minor strokes’ (a National Institutes of Health Stroke Scale [NIHSS] score below 6) are not conclusively proven to benefit from EVT and were therefore not included in the eligible population.^9^ Whilst the HERMES meta-analysis applied a cut-off of NIHSS ≤ 10 (showing a strong trend towards benefit but without statistical significance), there was no evidence of heterogeneity in treatment effect by NIHSS. However, individual trials have shown benefit from EVT with an NIHSS of 6 or more: ESCAPE (Endovascular Treatment for Small Core and Anterior Circulation Proximal Occlusion with Emphasis on Minimizing CT to Recanalization Times)^3^ and SWIFT PRIME (Solitaire FR With the Intention for Thrombectomy as Primary Endovascular Treatment for Acute Ischemic Stroke)^4^; and NIHSS of 8 or more: REVASCAT (Randomized Trial of Revascularization With Solitaire FR Device Versus Best Medical Therapy in the Treatment of Acute Stroke Due to Anterior Circulation Large Vessel Occlusion Presenting Within 8 Hours of Symptom Onset).^5^ Only MR CLEAN (Multicenter Randomized Clinical Trial of Endovascular Treatment for Acute Ischemic Stroke in the Netherlands)^1^ specifically enrolled patients with NIHSS below 6 and failed to show statistically significant benefit from EVT in the subgroup with NIHSS 2–15. Taking account of these data, we applied an NIHSS cut-off of 6 aligning with the three trials that included the largest numbers of patients in the NIHSS range 6–10.

The STOP-Stroke study^12^ reported that 20% of LAO strokes had an NIHSS of less than 6 (decision-point C). This was reinforced by El Tawil et al.^13^ These proportions give an estimate of 26,590 moderate/severe stroke patients (NIHSS 6 or more) with LAO in the UK annually.

### Time of onset and eligibility

Eligible stroke patients were defined as those with a known stroke onset time of less than 12 h before presentation or were Stroke with Unknown Time of Onset (SUTO) with a Last Seen Well (LSW) time within 12 h. No recent published thrombectomy trial has included patients beyond this time period.

A distribution of presentation times was derived from SNNAP^10^ but this was not reported by stroke severity. Stratification by severity was performed using service level SSNAP data for the calendar year 2015 from a single large UK acute stroke unit (Northumbria Healthcare NHS Foundation Trust: 900 admissions annually) and for three years from a second unit (Royal Devon and Exeter NHS Foundation Trust: 700 admissions annually), which showed that 78% of stroke patients with NIHSS of 6 or more presented within 12 h of onset. The SSNAP figure for all stroke cases presenting within 12 h was lower at 55% which is consistent with Northumbria and Devon and Exeter data if later presentation of milder cases is accounted for. For the remaining 22% of patients with NIHSS of 6 or more, SNNAP data enabled estimation of the relative proportions presenting with (a) SUTO but LSW within 12 hours (68.5%) and (b) a known onset time greater than 12 h (31.5%; Figure 1, decision-point E).

According to SSNAP^10^ data, 81% patients present with a known time of onset, of whom 60% are within 4 h and 21.1% between 4 and 12 h (with 18.9% after 12 h). Therefore, the split between those presenting within 4 h and those between 4 and 12 h is 74% and 26%, respectively (Figure 1, decision-point F). After exclusions for onset time, stroke type, severity and location, the decision tree contains two cohorts of patients potentially eligible for EVT: ‘early presenters’ – i.e. those presenting within 4 h (mostly eligible for IVT within 4.5 h) and ‘late presenters’ – those ineligible for IVT because either their stroke onset was 4–12 h ago, or they were SUTO but LSW within 12 h. At this point in the decision tree, approximately 24,750 (25%) of stroke admissions are potentially eligible for EVT (9400 + 15,350). It was assumed that only ‘early presenters’ would be able to receive EVT treatment within 6 h of onset.^9^ Trial data indicate that from arrival at thrombectomy centre to arterial puncture it will take >60 min on average to groin puncture and at least another 45 min for recanalisation to be achieved.^14^ In addition, the majority of UK patients will require secondary transfer for EVT after initial local assessment. For late presenting patients (arrive beyond 4 h post onset), it was assumed that IVT would not be used. From this point in our decision tree, the two groups (Figure 1, decision-points G and H) are differentially influenced by application of AI.

### Clinical and radiological exclusions amongst the IVT eligible population

The largest group eligible for EVT were those early presenters i.e. 13,770 (14% of all stroke admissions). Further EVT exclusions associated with little prospect of successful reperfusion were a CT ASPECTS (Alberta Stroke Programme Early CT Score)^15^ of less than 6 or visible infarction of more than one-third of the middle cerebral artery (MCA) territory, and a pre-stroke mRS of 3 or more. As only 1.6% of the HERMES patients had an mRS of 3 or more, this group are excluded as EVT benefit is unproven. The STOP-Stroke study^12^ identified 8.7% of LAO stroke patients with a pre-stroke mRS of 3 or more, which is not dissimilar to reports from the study logs of trials included in HERMES.^9^

The HERMES meta-analysis reported that an ASPECTS of 0–5 did not demonstrate a statistically significant treatment benefit (odds ratio [OR] 1.24, 0.62–2.49)^9^ possibly because numbers in this category were small (9%). In contrast, clear benefit for EVT was demonstrated with a presentation ASPECTS score of 6–8 and 9–10. To estimate the differential impact on outcome of early radiological changes, we applied a post hoc analysis of the Interventional Management of Stroke (IMS)-3 trial CTA positive subgroup data,^14^ which reported LAO on CTA in 40/282 participants (14%) with ASPECTS 0–4 and 88/282 (31%) with ASPECTS 5–7. We allocated these proportions equally to each ASPECTS score, yielding an estimated proportion of almost 25% for ASPECTS 0–5 in proven LAO. A pre-stroke mRS of 3 or more, and/or ASPECTS of 0–5 would, therefore, exclude approximately 34%. It was assumed that no overlap exists between these two criteria as we were unable to identify any reports of an association between pre-stroke disability and the severity of early ischemic changes assessed by ASPECTS or any other method. Therefore, amongst the early presenting IVT eligible population, we estimated that 10% of total stroke admissions were eligible for EVT, before any AI exclusions. This equates to 10,130 patients per year (Figure 1, decision-point G).

Various modes of AI (CT-CTP, CTA-CS combined with ASPECTS, or MRI) have been proposed for the exclusion of patients with a large core infarct. Data from the EXTEND-IA (Extending the Time for Thrombolysis in Emergency Neurological Deficits–Intra-Arterial)^2^ trial and the Sistema Online d'Informació de l'Ictus Agut (SONIIA)^16^ Registry suggest that AI excludes a further 5% of those early presenters with moderate/severe LAO stroke and pre-stroke mRS below 3 because they have a large volume core and small penumbra. If optional AIs were used in the early presenting group, the decision tree shows that a further 500 patients would be excluded, leaving an EVT eligible population of 9620 patients, before any recanalisation (Figure 1, decision-point I).

### Clinical and radiological exclusions amongst the late presenting/SUTO population ineligible for IVT

In the group presenting with SUTO but LSW within 12 h, or with a known onset time between 4 and 12 h, information about EVT eligibility is less robust and reliant upon variable AI protocols. Within our population of moderate-to-severe ischemic strokes with LAO, we estimate 5390 would have a known time of onset between 4 h to 12 h. We also estimated from SSNAP that a population of 4010 would be LSW within 12 h, giving a population of 9400 in whom AI might identify salvageable brain tissue, the majority of whom would also have a pre-stroke mRS below 3 (Figure 1, decision-point H).

To identify the proportion of this group excluded by imaging, data from SWIFT^17^ and IMS-3^18^ trials were used. At baseline, 25% had an ASPECTS below 6. Furthermore, by comparing ASPECTS at baseline to follow-up (mostly at 24 h), 48% deteriorated from good to poor ASPECTS.^16^ It is assumed that this deterioration represented core infarct extension occurring within 12 h. Therefore, in total 73% of ‘late-presenting’ patients are excluded by an ASPECTS below 6 on initial CT. Clinical mRS exclusions (as in the early-presenting group) would exclude another 8%^12^ or 203 (of the remaining 2538 late-presenting patients with an ASPECTS score indicative of limited acute ischaemic damage), leaving a total of 2340 of 9400 eligible for AI (Figure 1, decision-point H). That is 75% of 9400 are excluded.

Data from the CTP group in MR CLEAN^19^ indicate that 43% had a large core of greater than 70 mL (using the definition applied in EXTEND^2^ and SWIFT-PRIME^4^ trials). Applying this proportion means that 1330 of the group remained definitely eligible for EVT (i.e. they had a smaller core and a larger volume of salvageable penumbral tissue; Figure 1, decision-point K).

### Recanalisation prior to EVT

Our estimates identify *9620 or 10,920* patients eligible for EVT, depending upon whether AI is used to identify salvageable brain tissue in those early presenters. A small proportion of these patients will recanalise spontaneously or in response to IVT before EVT is performed. The HERMES trials indicate that this occurred in 5% of those receiving IVT. Spontaneous recanalisation among patients not receiving IVT is estimated at 2% based on expert consensus (PW, GF, MJ), and the finding from the PROACT-II trial (Prolyse in Acute Cerebral Thromboembolism II),^20^ in which 2% of patients in the placebo arm had TIMI 3 (Thrombolysis in Myocardial Infarction rating scale [in which three represents complete recanalisation]); in this context, any recanalisation that is less than complete would not exclude EVT. Thus recanalisation prior to EVT excludes 510 patients (480 if the AI pathway followed) from the early presenting population presenting (Figure 1, decision-points J). Spontaneous recanaliation would exclude 30 patients from the late presenting/SUTO group (Figure 1, decision-point L).

### Sensitivity analyses

Results of sensitivity analyses are shown in Table 1. For LAO, we identified retrospective study extremes between 13%^21^ and 88%,^22^ which were regarded as unreliable for modelling. More robust data from a prospective cohort reported a lower LAO estimate of 33%,^23^ and the EXTEND-IA^2^ screening log-identified LAO in 53% of IVT-eligible patients, so these data were used as the basis for a 30–50% range of LAO incidence. In the absence of other credible data sources, a pragmatic 10% range was also used for exclusion by onset time, ASPECTS, mRS and the proportion excluded due to a large core. The LAO proportion and the numbers of patients presenting with a known onset time within 12 h, had the greatest impact on the estimates of eligibility.

**Table 1.** Univariate sensitivity analyses.

Decision point	Value (%)	Eligible population
Proportion of LAO strokes		
High value	50	12,030–13,670
Low value	30	7220–8200
Proportion of moderate/severe strokes presenting early		
High value	88	10,860–12,100
Low value	68	8390–9860
Proportion of late-presenting patients excluded by ASPECTS and mRS of 3 or more		
High value	65	9620–11,460
Low value	85	9620–10,410
Proportion of late-presenting patients with large core		
High value	33	9620–11,170
Low value	53	9620–10,710

ASPECTS: Alberta Stroke Programme Early CT Score; LAO: large artery occlusion; mRS: modified Rankin Scale.

## Discussion

Based on the available evidence from intervention trials and prospective registries in EVT, we estimate 9140–10,920 patients in the UK with acute ischemic stroke are eligible for EVT annually i.e. approximately 10% of strokes admitted to hospital. This is consistent with other reports. Chia et al.^22^ estimated a range of 7–13% for EVT eligibility presenting to two of three Australian hyper-acute stroke sites serving a population of approximately 150,000. The lower bound of our estimate is defined by restricting EVT only to those early presenters (9620/year). The upper bound is defined by the inclusion of all early-presenting patients without the use of AI (9620/year) to which are added those late-presenting patients with a favourable imaging profile (1310/year). AI would exclude around 5% (500/10,130) of early-presenting and otherwise eligible patients from EVT but would include around 56% (1310/2350) of late-presenting (IVT-ineligible) patients as eligible for EVT. Thus, although the overall requirement (eligibility) for EVT is relatively unchanged by AI, its use would affect EVT treatment decisions in approximately 15% (1810/12,470) of otherwise eligible patients.

Where possible our decision points are based upon the large prospective SSNAP registry, which covers the UK excluding Scotland. Case ascertainment by SSNAP in England (population 55 million) exceeds 98%. SSNAP or randamised controlled trials (RCTs) data provide the main evidence criterion for 9 of the 12 decision points. The main uncertainties are in the smaller group of late-presenting patients with LAO and NIHSS greater than 6, for whom limited high-quality data are available around eligibility for EVT (decision-point H) since this population was the least represented in the trials. However, this group is small and sensitivity analyses show that changing assumptions have little impact upon model outcomes.

The proportion of patients considered appropriate for EVT is dependent upon the frequency of LAO, but previous reports vary. Amongst the recent thrombectomy trials which reported screening and eligibility data, the rate of LAO was 53% in EXTEND-IA^2^ and 48% in SWIFT PRIME.^4^ Rai et al.^21^ estimated the incidence of LAO from a retrospective sample of nearly 3000 patients referred to a tertiary-level academic hospital in West Virginia, over 90% of whom had CTA, with LAO demonstrated in only 12%. However, complete case ascertainment is uncertain as many patients were secondary transfers, and over 70% of LAO were M1 occlusions. Smith et al.^12^ identified, after expert review, an LAO rate of 46% in patients with confirmed stroke referred to two large academic US centres, using a broader definition which included the anterior and posterior cerebral arteries, and second-order branches (so M2). A recent prospective study in the UK identified an LAO rate of 39%.^13^ Our rate of LAO at 40% may be a small overestimate, but we consider this to be based on the most reliable information available.

The selection of patients by AI based upon current best evidence had relatively little effect on the overall numbers eligible for treatment but altered the eligibility decision in 15% of cases. The impression that a relatively small proportion of early-presenting patients with LAO on CTA would be subsequently ruled out by AI (5% in our model) is corroborated by EXTEND-IA^2^ trial. The results from the DAWN trial (NCT02142283) will be valuable for clarifying the proportion of patients with an unknown symptom onset time who should be offered EVT according to AI.

With no formally commissioned services, the UK is starting from a low baseline; in 2017, NHS England anticipates funding treatment of 1000 patients in the first year of formal commissioning. The midpoint of our estimate for a UK population suitable for EVT (10.8% of all stroke admissions) combined with the absolute benefits estimated in a recent individual patient data meta-analysis^24^ suggest that EVT with national coverage could achieve an additional 2420 patients with independent functional outcomes, or as many as 4280 patients (4% all stroke admissions) with a reduced level of disability compared to IVT alone. Implicit in this estimates is the assumption that outcomes for posterior circulation EVT (which are included in our estimates of eligible population) are the same as those for anterior circulation EVT. There is an absence of evidence about posterior circulation EVT, but in light of outcomes for basilar artery occlusions treated with IVT, we judge this assumption reasonable at this time. Based on a range of estimates, the mean monthly cost to the UK National Health Service and social care providers of caring for people who lose their independence because of stroke (an mRS of 3, 4 or 5) was estimated at £790 (US$1,300/€980 at 2014 exchange rates) at 2013–2014 prices.^25^ Assuming 2420 people would maintain independence because of EVT, the savings (before costs for EVT are included) over 12 months post-stroke are greater than £22 million (US $36 million/€27 million at 2014 exchange rates). A cost-effectiveness analysis from the US^26^ reported that EVT is a highly cost-effective intervention in the prevention of stroke-related disability with an incremental cost-effectiveness ratio of $3,000 per quality-adjusted life year (QALY). A more recent study projected that EVT dominated thrombolysis alone when future savings from reduced social care need were included, and despite the higher costs of providing EVT, there was a saving of £30,000 over a patient’s lifetime to health and social care providers and before the consequences of lost productivity in the working age stroke population were accounted for.^27^ This equates each year in the UK, to a net realisable saving of £73 million each year over patient’s lifetimes.

## Conclusion

Between 9620 and 10,920 stroke patients per year in the UK could be eligible for EVT based on current level-1 evidence, which approximates to 10% of stroke admissions. Given the magnitude of the potential clinical and wider economic benefits from EVT, it should now be a key priority to address the substantial infrastructure and workforce obstacles impeding rapid and widespread implementation.
